# Means of increasing response rates in a Norwegian dietary survey among infants – results from a pseudo-randomized pilot study

**DOI:** 10.1186/s12874-019-0789-6

**Published:** 2019-07-09

**Authors:** Jannicke Borch Myhre, Lene Frost Andersen, Kristin Holvik, Helene Astrup, Anne Lene Kristiansen

**Affiliations:** 1 Department of Nutrition, Institute of Basic Medical Sciences, Faculty of Medicine, University of Oslo, PO Box 1046, Blindern, 0317 Oslo, Norway; 2 Department of Chronic Diseases and Aging, Norwegian Institute of Public Health, PO Box 222, Skøyen, 0213 Oslo, Norway

**Keywords:** “Response rate”, “Incentive”, “Monetary incentive”, “Lottery”, “Personalization”, “handwritten name and address”, “Printed label”, “E-mail invitation”, “Postal invitation”, “Dietary survey”

## Abstract

**Background:**

Postal surveys are widely used in scientific studies, including dietary surveys, but few studies about methods to increase participation in national dietary surveys are published. In the present study we compared response rates in a pilot study to a national dietary survey among infants using two different incentives (gift certificate or lottery), personalization in the form of handwritten name and address vs. a printed label and mode of sending out invitations (e-mail or postal invitation).

**Methods:**

In this parallel-design pseudo-randomized pilot trial, a nationally representative sample of 698 mothers of infants aged 6 and 12 months was drawn from the Norwegian National Registry and invited to complete a food frequency questionnaire about their infant’s diet. One half of the mothers of 6 month olds were randomized by alternation to the lottery group (*n* = 198) and offered to participate in a lottery of two prizes (500 EUR and 1000 EUR). The other half (*n* = 200) was offered a gift certificate (50 EUR) upon completion of the questionnaire. Each incentive group was randomized by alternation to receiving an invitation with handwritten name and address or a printed label. For the mothers of infants aged 12 months (*n* = 300), 150 mothers received an e-mail invitation and 150 mothers received a postal invitation. Logistic regression was used for testing differences between the groups.

**Results:**

The response rate was significantly higher (*p* = 0.028) in the gift certificate group (72%) than in the lottery group (62%). No difference was seen between those receiving an invitation with a handwritten name and address (68%) compared to a printed label (66%, *p* = 0.72). A somewhat higher response rate was seen when using the postal (50%) compared to the e-mail invitation (43%, *p* = 0.25).

**Conclusions:**

In this pseudo-randomized parallel-design trial of women participating in a national dietary survey among infants, the response rate was higher when offered a gift certificate than when participating in a lottery. Handwritten name and address did not affect participation compared to a printed label. Only a moderate difference was seen between the postal and e-mail invitation. Others conducting similar methodological studies are encouraged to publish their results to expand the knowledge basis in this area.

## Background

Postal surveys are widely used for collecting data from a large number of participants in scientific studies, including dietary surveys [1–3]. Advantages of using postal surveys include being relatively low cost compared to other study designs [4], providing the possibility to reach persons over a large geographical area as well as allowing the respondent to answer questions without having to face an interviewer. A major concern when using postal invitations is the problem of low response rates. Low response rates are likely to impair the validity of study findings due to non-response bias [5], meaning that those who participate and those who choose not to participate differ in aspects relevant to the study outcomes [6]. Several approaches have been tested to increase response rates in studies using postal questionnaires, and the use of monetary incentives has often been shown to increase response rates [7, 8]. Other manners of improving response rates have also been tested, such as increasing the saliency of the invitation, shortening of the questionnaire, increasing personalization and various follow-up strategies [7]. In national dietary surveys an important aim is to describe the diet of the general population and results are not likely to be representative of the population as a whole if only a small portion of those who are invited complete the survey. However, to our knowledge, only one previous study looking at different strategies to increase response rates in national dietary surveys has been published [9]. This was a study on adults participating in the first Norwegian national dietary survey (Norkost 1993/94) where a higher response rate was seen in the group offered to participate in a lottery compared to the group not offered an incentive.

In Norway, the third national dietary survey among infants aged 6 and 12 months (Spedkost 3) was carried out in 2018 and 2019. As response rates in dietary surveys have shown a tendency to decrease over the past decades [5], a pilot study with special emphasis on obtaining acceptable response rates was carried out prior to the main study. We aimed to study the effect on response rates of two monetary incentives, namely participation in a lottery or offering a gift certificate to all who completed the study. We also aimed to investigate whether personalization in the form of using a handwritten name and address on the envelope with the invitation would affect participation compared to a printed label. Lastly, we wanted to study if receiving an e–mail invitation resulted in a different response rate than receiving a postal invitation.

## Methods

### Sample and design

The study was performed as a pseudo-randomized parallel-design trial. Data collection was carried out in Norway from September to November 2017. As the present study was a pilot study to the next Norwegian national dietary survey in infants aged 6 and 12 months, both infants aged 6 and 12 months were included also in the pilot study. While the main study is a longitudinal study assessing dietary habits in the same infants at 6 and 12 months of age, the pilot studies included two separate samples of infants aged 6 and 12 months to reduce the length of the study period. Financial restraint caused the largest possible number of participants in the pilot study to be 1000 pairs of infants/mothers. Of these, 400 were determined to be infants aged 6 months participating in the testing of the effect of incentive type and personalization of the invitation. The remaining 600 pairs of mothers/infants were infants aged 12 months. As the pilot study also included a calibration study of the web based and the paper based version of the study questionnaire, 300 infant/mother pairs of the sample of 12 month olds were invited to the calibration study and 300 infant/mother pairs were invited to the testing of response rate according to mode of invitation. The results from the calibration study will not be reported herein.

**Fig. 1:**
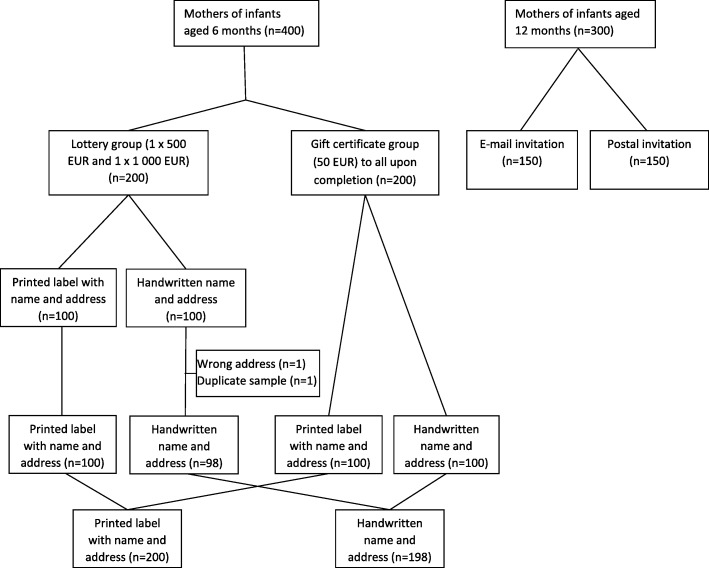
Flow chart of study design

The nationwide sample of infants aged 6 months included 400 infants born in the period from March 1st to March 5th 2017, while the sample of infants aged 12 months included 300 infants born in the period from September 1st to September 7th 2016. Both samples were drawn from the Norwegian National Registry, the two groups being 6 and 12 months of age at the time of invitation. Only infants born to mothers who themselves were born in Norway, Sweden or Denmark were included in the drawn sample; also, a cell phone number had to be registered on the mother for the mother and infant to be included. The mother’s telephone number was added from an external commercially available database. About 65% of women of childbearing age had a registered cell phone number in this database. Reasons for not being registered in the database could for instance be having a cell phone registered at the work place, having a secret telephone number or the cell phone being registered on other members of the family. Only one infant could participate from each household, so for mothers giving birth to twins or triplets, only one of the infants was invited to participate. The drawn sample from the National Registry included information about the name and address of the mother and infant, the birth number of the infant, the gender of the infant and the mother’s year of birth. For the infants aged 6 months, the received sample was sorted according to the infant’s birth number. The sample was then sorted according to postal zip code to ensure similar geographical distribution and the invitees were randomized by alternation to either being offered a gift certificate of 500 NOK (50 EUR) upon completion of the questionnaire (gift certificate group) or being offered participation in a lottery of two gift certificates, one with a value of 5000 NOK (500 EUR) and one with a value of 10,000 NOK (1000 EUR) (lottery group). The invitees in each of these groups were then randomized by alternation to either receive an invitation with a handwritten name and address or an invitation with a printed label (Fig. 1). All randomizations were performed by the first author (JBM).

For the infants aged 12 months, one half of the mothers received a postal invitation, while the other half received an e-mail invitation (Fig. 1). To obtain the invitees’ e-mail addresses the full birth number of the mother had to be obtained from the National Registry. This information was provided for mothers of the first 150 infants in the list sorted according to the infant’s birth number and therefore these individuals constituted the group receiving an e-mail invitation. The consecutive 150 infants constituted the group receiving a postal invitation. E-mail addresses were collected from the Norwegian Agency for Public Management and eGovernment (Difi). All mothers to infants aged 12 months were offered a gift certificate of 300 NOK (30 EUR) as incentive upon completion of the survey. The amount used as monetary incentive among the infants aged 12 months was somewhat lower than the incentive among the infants aged 6 months due to economic constraints.

For all the invitees, the invitation contained information about the study and a link to a web based questionnaire. The invitation informed the participants that the purpose of the study was to test the feasibility of the study design and the questionnaire, but it did not specifically include information about the additional purpose of testing the effect of different incentives and invitation modes on response rates.

Approximately 1 week after receiving the invitations, all non-responders were contacted by telephone by a project assistant to clarify questions about the study and motivate to participate. Those invited who still had not responded within 3 weeks of receiving the invitation received a written reminder including a paper based version of the questionnaire. For the infants aged 6 months, those who received invitations with handwritten name and address the first time also received a reminder with handwritten name and address. Likewise, those who received invitations with a printed label also received a reminder with a printed label. For the infants aged 12 months, all the postal invitations and reminders had handwritten name and address.

The Norwegian Centre for Research Data approved the study (project number 53936).Written informed consent was obtained from the mother/parents. The written consent was included as the first question of the questionnaire “Select “yes” below if you consent to participating in the study. If you do not wish to participate and do not wish to receive a phone call or a written reminder, please select “No” (two options below; Yes and No)”.

### Questionnaires

The questionnaires aimed to describe the infant’s feeding practices at 6 and 12 months of age, and included questions about breastfeeding, introduction of complementary foods and dietary supplements in addition to some background questions about the infant’s parents such as parental education and the mother’s employment situation prior to giving birth, family situation and smoking habits. The questionnaires also asked for the weight and length of the infant at birth and at 6 or 12 months of age. More information about similar previous versions of the questionnaires is available in Kristiansen et al. (2010 and 2013) [3, 10]. For the infants aged 6 months the questionnaire comprised 15 pages (in the paper based version) containing a total of 28–127 questions (the number of questions depended on previous choices when answering the questionnaire) and took about 20 min to complete. For the infants aged 12 months, the questionnaire asked about a higher variety of foods and comprised 23 pages (in the paper based version) containing a total of 247–275 questions (depending on the participant’s answers). The questionnaire for the infants aged 12 months took about 40 min to complete.

### Gift certificates

As it is not permitted to send money in regular mail in Norway, gift certificates were used as incentives in the present study. The gift certificates were generic and could be used in more than 5000 stores all over Norway.

### Statistical analysis

Statistical analyses were carried out using the statistical software package IBM SPSS Statistics version 25. All tests were two-sided, and a significance level of 0.05 was chosen. Differences in response rates between the two incentive groups, between the use of a label or handwritten address and between sending out a postal or an e-mail invitation were tested with logistic regression and results are presented as odds ratios (OR) and 95% confidence intervals (CI). Logistic regression was also used for checking for possible interactions between incentive type and handwritten vs. labeled name and address on the envelope, and for adjusting for incentive type when estimating the effect of handwritten vs. labeled name and address on response rates.

For the background variables including the infant’s gender, geographical region, mother’s age, mother’s employment prior to birth, parent filling in the questionnaire and parents’ educational level, the proportion of participants in each category of the variable is presented. Differences in proportions of participants belonging to the various background characteristics groups according to incentive group, receiving a label or handwritten address and the groups receiving a postal or an e-mail invitation were tested using the chi-square test. When obtaining a significant chi-square result in a table larger than 2 × 2, adjusted standardized residuals were calculated to identify the cells with the largest deviations from expected values (a cut-off of +/− 1.96 was used) [11]. The adjusted standardized residuals provide a measure of the strength of the difference between observed and expected values and show how significant each cell is to the obtained chi square value. If the adjusted standardized residual is greater/ less than +/− 1.96, the cell’s observed frequency is less/greater than the expected frequency [11].

## Results

### Response rate in the dietary survey of infants aged 12 months

One infant aged 6 months was sampled twice and one letter was returned by the postal service due to an unknown address. Hence, the final eligible sample of 6 month olds was 198 invitees for the lottery group and 200 invitees for the gift certificate group.

The response rate was significantly higher in the gift certificate group (72%) compared to the lottery group (62%, OR for participation 1.6 (95% CI 1.1, 2.4), *p* = 0.028) (Table 1). No difference in participation was seen between those receiving an invitation letter with a handwritten name and address (68%) and those receiving an invitation letter with a printed label (66%, OR 1.1 for participation (95% CI 0.7, 1.6) *p* = 0.72) (Table 1). This was not altered when controlling for incentive type (lottery or gift certificate) (OR for participation 1.1 (95% CI 0.7, 1.6), *p* = 0.73), and there was no statistical interaction between the incentive type and the manner of writing name and address on the envelope (*p* = 0.45).

**Table 1:** Response rates and odds ratios (OR) with 95% confidence intervals for participation according to incentive and manner of writing name and address on envelope, 6 month olds (*n* = 398)

	Participated/invited (response rate)	OR (95% CI)	p^a^
Incentive		1.6 (1.1, 2.4)	0.028
Gift certificate^b^	144/200 (72%)		
Lottery^c^	122/198 (62%)		
Personalization		1.1 (0.7, 1.6)	0.72
Handwritten name and address^d^	134/198 (68%)		
Printed name and address^e^	132/200 (66%)		

^a^Differences in response rates between the groups were tested with logistic regression

^b^All participants received a gift certificate worth 500 NOK (50 EUR)

^e^Participants took part in a lottery of two prizes; 5000 NOK (500 EUR) and 10,000 NOK (1000 EUR)

^d^Invited sample received invitation envelope with a handwritten name and address

^e^Invited sample received invitation envelope with name and address on a printed label

No significant differences were seen in the distribution of background characteristics between the participants in the lottery group and the gift certificate group (Table 2). When comparing background characteristics of the participants in the group receiving an invitation letter with a handwritten name and those receiving an invitation letter with a printed label, a significant difference was observed in the distribution across different age groups (*p* = 0.033, Table 2). However, inspection of the adjusted standardized residuals showed that no cells had values larger than +/− 1.96 (maximum/minimum values were 1.89 and − 1.89 for the oldest age group).

**Table 2:** Background characteristics of the participants, 6 month olds (*n* = 266)

	Lottery^a^ (*n* = 122)	Gift certificate^b^ (*n* = 144)	P^c^	Handwritten^d^ address (*n* = 134)	Label address^e^ (*n* = 132)	P^c^
Infant’s gender, n (%)			0.98			0.22
boy	62 (51%)	73 (51%)		63 (47%)	72 (55%)	
girl	60 (49%)	71 (49%)		71 (53%)	60 (45%)	
Geographical region, n (%)			0.92			0.99
Oslo/Akershus (capital region)	28 (23%)	34 (24%)		30 (22%)	32 (24%)	
Hedmark/Oppland	10 (8%)	10 (7%)		10 (7%)	10 (8%)	
South-Eastern Norway	15 (12%)	24 (17%)		19 (14%)	20 (15%)	
Agder/Rogaland	21 (17%)	19 (13%)		19 (14%)	21 (16%)	
Western Norway	23 (19%)	25 (17%)		26 (19%)	22 (17%)	
Trøndelag	13 (11%)	18 (13%)		17 (13%)	14 (11%)	
Northern Norway	12 (10%)	14 (10%)		13 (10%)	13 (10%)	
Mother’s age, years, n (%)			0.53			0.033
20–25	6 (5%)	11 (8%)		5 (4%)	12 (9%)	
26–30	50 (41%)	60 (42%)		60 (45%)	50 (38%)	
31–35	41 (34%)	52 (36%)		40 (30%)	53 (40%)	
≥36	25 (20%)	21 (15%)		29 (22%)	17 (13%)	
Employment prior to birth, mother, n (%)			0.09			0.81
Employed (full/part time)	113 (93%)	124 (86%)		120 (90%)	117 (89%)	
Not working	9 (7%)	20 (14%)		14 (10%)	15 (11%)	
Who filled in the questionnaire, n (%)			0.26			0.12
Mother	114 (93%)	129 (90%)		126 (94%)	117 (89%)	
Father/mother and father	8 (7%)	15 (10%)		8 (6%)	15 (11%)	
Mother’s educational level, n (%)			0.77			0.46
High school or lower	35 (29%)	39 (27%)		40 (30%)	34 (26%)	
College/university	87 (71%)	105 (73%)		94 (70%)	98 (74%)	
Father’s educational level, n (%)^f^			0.17			0.94
High school or lower	63 (53%)	63 (44%)		64 (48%)	62 (48%)	
College/university	57 (48%)	80 (56%)		69 (52%)	68 (52%)	

^a^Participants took part in a lottery of two prizes; 5000 NOK (500 EUR) and 10,000 NOK (1000 EUR)

^b^All participants received a gift certificate worth 500 NOK (50 EUR)

^c^Differences in percentages between the two incentive groups and between the two manners of writing name and address and postal or e-mail invitation were tested with the chi-square test

^d^Invited sample received invitation envelope with a handwritten name and address

^e^Invited sample received invitation envelope with name and address on a printed label

^f^Three participants not included due to missing information about father’s education

### Response rate in the dietary survey of infants aged 12 months

A somewhat higher response rate was seen in the group receiving the postal invitation (50%) compared to those receiving an e-mail invitation (43%), but the difference was not statistically significant (OR for participation 1.3 (95% CI 0.8–2.1), *p* = 0.25) (Table 3).

**Table 3:** Response rates and odds ratios (OR) with 95% confidence intervals for participation according to mode of invitation, 12 month olds (*n* = 300)

	Participated/invited (response rate)	OR (95% CI)	p^a^
Mode of invitation		1.3 (0.8, 2.1)	0.25
Postal invitation	75/150 (50%)		
E-mail invitation	65/150 (43%)		

^a^Differences in response rates between the groups were tested with logistic regression

Concerning background characteristics of participants in the groups receiving an e-mail invitation or postal invitation, respectively (Table 4), a tendency towards a lower educational level was seen in the group participating after receiving a postal invitation, but this difference was not statistically significant (*p* = 0.08 for mother’s education and 0.09 for father’s education). No statistically significant differences were seen for the other background characteristics (Table 4).

**Table 4:** Background characteristics of the participants, 12 month olds (*n* = 140)

	E-mail invitation (*n* = 65)	Postal invitation (*n* = 75)	p^a^
Infant’s gender, n (%)			0.65
boy	38 (58%)	41 (55%)	
girl	27 (42%)	34 (45%)	
Geographical region, n (%)			0.42
Oslo/Akershus	10 (15%)	19 (25%)	
Hedmark/Oppland	7 (11%)	8 (11%)	
South-Eastern Norway	13 (20%)	12 (16%)	
Agder/Rogaland	12 (18%)	8 (11%)	
Western Norway	13 (20%)	11 (15%)	
Trøndelag	5 (8%)	5 (7%)	
Northern Norway	5 (8%)	12 (16%)	
Mother’s age, years, n (%)			0.27
20–25	4 (6%)	11 (15%)	
26–30	20 (31%)	27 (36%)	
31–35	24 (37%)	23 (31%)	
≥36	17 (26%)	14 (19%)	
Employment prior to birth, mother, n (%)^b^			0.31
Employed (full/part time)	59 (91%)	63 (85%)	
Not working	6 (9%)	11 (15%)	
Who filled in the questionnaire, n (%)			0.49
Mother	58 (89%)	64 (85%)	
Father/mother and father	7 (11%)	11 (15%)	
Mother’s educational level, n (%)^b^			0.08
High school or lower	10 (16%)	21 (28%)	
College/university	54 (84%)	54 (72%)	
Father’s educational level, n (%)^c^			0.09
High school or lower	28 (44%)	43 (58%)	
College/university	36 (56%)	31 (42%)	

^a^Differences in percentages between the two invitation modes were tested with the chi-square test

^b^Information was missing for one participant (*n* = 139)

^c^Information was missing for two participants (*n* = 138)

## Discussion

The results from this pseudo-randomized trial showed that the response rate among mothers of infants aged 6 months was 10 percentage points higher when offered a gift certificate of 500 NOK (50 EUR) upon completion, compared to being offered participation in a lottery of two gift certificates, one with a value of 5000 NOK (500 EUR) and one with a value of 10,000 NOK (1000 EUR). This difference was statistically significant. The response rates did not differ according to whether the invitees received an invitation envelope with handwritten name and address compared to a printed label. For the mothers of the infants aged 12 months, a 7 percentage points higher response rate was seen in the group receiving a postal invitation compared to those receiving an e-mail invitation, but the difference was not statistically significant.

In Norway, two nationally representative dietary surveys in infants aged 6 and 12 months have been carried out previously, the first one in 1998/1999 [12, 13] and the second one in 2006/2007 [3]. In both previous studies the invitations were sent out using regular mail and the participants were offered to take part in a lottery of monetary prizes upon completion of the questionnaire. In 1998/1999, 80% of the invited sample participated when the infant was 6 months old [12], while the response rate had fallen to 67% in 2006/2007 [3]. We were therefore quite surprised and very pleased to achieve a response rate of more than 70% in the gift certificate group in the present study. In addition to the effect of the gift certificate, the fact that most mothers were still on maternity leave when receiving the invitation, the relatively short questionnaire, the possibility to answer either on the Internet or on paper and the topic of the questionnaire often being of interest to the parents may have been other factors contributing to the high response rate. Effort was also put into making the invitation brochure and paper version of the questionnaire look appealing.

In the present study, the lottery seemed to be a less effective incentive than the gift certificate. This was in spite of the participants being informed of how many participants that were invited to the lottery, showing a relatively high chance of winning (1/100) compared to many other lotteries. Hence, in spite of the relatively high chance of winning, the participants might still have found it unlikely that they would actually win and therefore were not as motivated by participating in the lottery. The gift certificate also seemed to cause more engagement amongst the participants than the lottery as we received several questions regarding when the gift certificates would arrive, while no participants contacted us with questions about the lottery. The use of monetary incentives is well documented to have an impact on response rates in general. In a Cochrane review from 2009, the odds of response to postal questionnaires were almost doubled with the use of monetary incentives [7]. Similar results were also found in a meta-analysis of randomized trials of monetary incentives and response rates from 2005 which concluded that monetary incentives increase mailed questionnaire response [8]. Likewise, a systematic review of recruitment strategies on general practitioner’s survey response rates showed that both monetary incentives and non-monetary incentives (such as scratch lottery tickets or a pen incentive) were more effective in increasing response rates compared to no incentive, with monetary incentives being somewhat more effective than non-monetary incentives [14]. In a Norwegian dietary survey among adults conducted in 1993/94 [9], the results showed that the response rate was higher amongst those offered to take part in a lottery (65%) compared to those who were offered no incentive (54%). A more recent study from New Zealand [15] using a postal questionnaire to describe factors influencing eating behavior and weight in women aged 40–50 showed that the response rate at baseline was significantly higher when receiving a small monetary token of NZ$ 5 (3 EUR) along with the invitation (76%) compared to not receiving the monetary token (64%).

In the present study gift certificates were used as a substitute for money, and it might be argued that using a gift certificate is not the same as offering real money. However, the gift certificates were generic and could be used in a wide range of stores; hence it is likely that many participants would view them as almost equal to receiving the actual amount in money.

Some previous studies have found that writing the name and address of the recipient on the envelope by hand might have a positive impact on response rates [16, 17]. In the previously mentioned Cochrane review published in 2009 [7], it was estimated that the odds of responding to a postal questionnaire was increased by 25% when using a handwritten name and address rather than a printed label. We theorized that this effect might be even stronger now than previously because it might be less common to receive letters with handwriting on the envelope now than before. However, no such effect was observed. Indeed, the postal invitation per se, regardless of whether name and address were handwritten, tended to yield a higher participation rate than the e-mail invitation, implying that just receiving a letter other than advertising may be special enough to catch the invited sample’s attention as fewer letters are sent by regular mail now than previously [18]. The difference in response rate between the postal invitation group and the e-mail invitation group was not statistically significant. Having conducted power analyses prior to study start would have been an advantage for the interpretation of the results. However, as power calculations are not recommended to be conducted a posteriori, this has not been done [19]. Hence, the lack of a significant difference between groups might have been caused by a too small sample size. When deciding the design for the main study, an evaluation of how large of a difference in response rates between the tested measures that would be of a real life interest was necessary. Some measures would be more expensive and/or labor intensive to make use of in the main study than others. Writing all names and addresses by hand would for instance have involved a substantially larger workload than using printed labels, while choosing regular mail over e-mail would only imply a smaller amount of extra work. While the difference in response rate of 7 percentage points between the e-mail and postal invitation groups was not significant, little extra effort was needed to send out invitations using regular mail compared to e-mail and hence this alternative was chosen for the main study.

Interestingly, in the present study the postal invitation seemed to produce a sample with a slightly higher proportion of mothers without a college/university education (28%) than the e-mail invitation (16%). This might be caused by the sample of invited mothers to the postal group by chance included more mothers without a college/university education, or that the postal invitation could have been more appealing to the mothers without higher education. Individuals without higher education are often more difficult to recruit to epidemiological studies [5], therefore this observation may be of particular interest.

Although monetary incentives are commonly used to motivate to study participation, their use may also raise ethical concerns [20]. Offering subjects money or other refunds for their participation could influence them to make financially-motivated choices against what they would otherwise feel sensible. In the present study participants randomized to the gift certificate group were offered a gift certificate of 500 NOK (50 EUR) upon completion of the study. This amount is equal to about 1.5 h of the Norwegian average hourly wage [21]. This amount is also commonly used when recruiting participants to various focus groups organized by commercial companies, for instance as an aid in product development [22]. In a commentary to the 13th guideline of the International Ethical Guidelines for Health-related Research Involving Humans prepared by the Council for International Organizations of Medical Sciences, it is stated that “Especially when the research poses low risks, providing compensation for participation should not raise concerns about undue inducement” [23]. Participation in the present survey involved low risk for the participants, and therefore providing compensation should not be a problem. It has also been argued that the problem of offering monetary incentives to research participants is rather that the participants receive too little for their effort than that they receive too much [24]. Low or no incentives for participation may lead to low response rates causing the results of the study to be of questionable, or even no value. This raises ethical questions as studies are often accepted by ethical committees because the knowledge that is generated is of such a value that it outweighs the potential risks for the participants. If the study cannot answer the research question due to an insufficient sample size, those who participated have invested their time and effort for limited or no benefit to society [25]. Families with small children often live quite busy lives and we saw it as reasonable to provide them with some compensation for investing their time in our study.

Strengths of the present study include the pseudo-randomized design, a nationally representative sample and the possibility to investigate both the effect of the incentives and handwritten name and address in the same participants.

Limitations include the lack of a control group receiving no incentive to see the effect of the lottery as such. However, this option was not included as it was expected that a non-incentive group would achieve the lowest response rate. Other researchers have found response rates to improve when including a monetary incentive in the invitation letter rather than handing out incentives to those who complete the survey [7, 15]. This would have been an interesting study arm to include in the study. Moreover, the group of infants aged 12 months was not truly randomized to receiving a postal invitation or an e-mail invitation as the first 150 infants in the list from the National registry (sorted according to the infant’s birth number) were the ones for whom we received a birth number for the mother. Hence, they constituted the e-mail group as full birth numbers were necessary to obtain e-mail addresses. However, we find it unlikely that this influenced the results to a large extent as there should be no reason to believe that the first 150 infants in the list would be different from the last 150 infants. They were only separated in age by a maximum of 1 week. Also the lack of power calculations before conducting the study is a limitation causing the study to be underpowered to detect modest differences in response rates between groups.

Our results apply foremost to women of childbearing age asked to complete a questionnaire about their infant’s diet. The results may not be directly generalizable to other population groups and other study designs.

As this was a pilot study, the results from the present study were incorporated into the main national dietary study in infants that was conducted during the fall of 2018 and the spring of 2019. In accordance with the findings reported herein, participants were offered a gift certificate worth 500 NOK (50 EUR) upon completion of the questionnaire and invitations were sent out using regular mail with name and address on a printed label.

Very few studies looking at methods to increase response rates in national dietary surveys were found when planning the present study, showing a large information gap in this area with a need for more research. It might be that pilot studies similar to ours are conducted prior to main studies in several countries, but that the results remain unpublished. Whenever possible, results should be published as they are of great interest to others planning similar studies.

## Conclusion

In this pseudo-randomized parallel-design trial of women participating in a Norwegian national dietary survey among infants, the response rate was higher when receiving a gift certificate upon completion of the study than when participating in a lottery. Receiving an invitation envelope with handwritten name and address did not affect participation compared to a printed label. A non-significant tendency towards a higher response rate was seen in the group receiving a postal invitation compared to the group receiving an e-mail invitation. These findings will be useful to others planning epidemiological studies, particularly in women of childbearing age. Other investigators conducting similar methodological studies are encouraged to publish their results to expand the knowledge basis in this area.

## Data Availability

The datasets analyzed during the current study are available from the corresponding author on reasonable request.

## Abbreviations

CI: confidence interval
Difi: Norwegian Agency for Public Management and eGovernment
EUR: Euro
NOK: Norwegian krone (Norwegian currency)
OR: odds ratio
